# Supplementary material for: Long-term isolation and introgression shape the genomic distinctiveness of Rice’s Whale
Source: bioRxiv. 2026 Jun 19:2026.06.15.732430. Preprint. [Version 1] doi: 10.64898/2026.06.15.732430 (PMC13308004; doi:10.64898/2026.06.15.732430)
Supplement: Supplement 7 [file NIHPP2026.06.15.732430v1-supplement-7.pdf]

bioRxiv preprint doi: https://doi.org/10.64898/2026.06.15.732430; this version posted June 19, 2026. The copyright holder for this preprint (which was not certified by peer review) is the author/funder, who has granted bioRxiv a license to display the preprint in perpetuity. It is made available under a CC-BY 4.0 International license.

Supplemental Figures

bioRxiv preprint doi: https://doi.org/10.64898/2026.06.15.732430; this version posted June 19, 2026. The copyright holder for this preprint (which was not certified by peer review) is the author/funder, who has granted bioRxiv a license to display the preprint in perpetuity. It is made available under a CC-BY 4.0 International license.

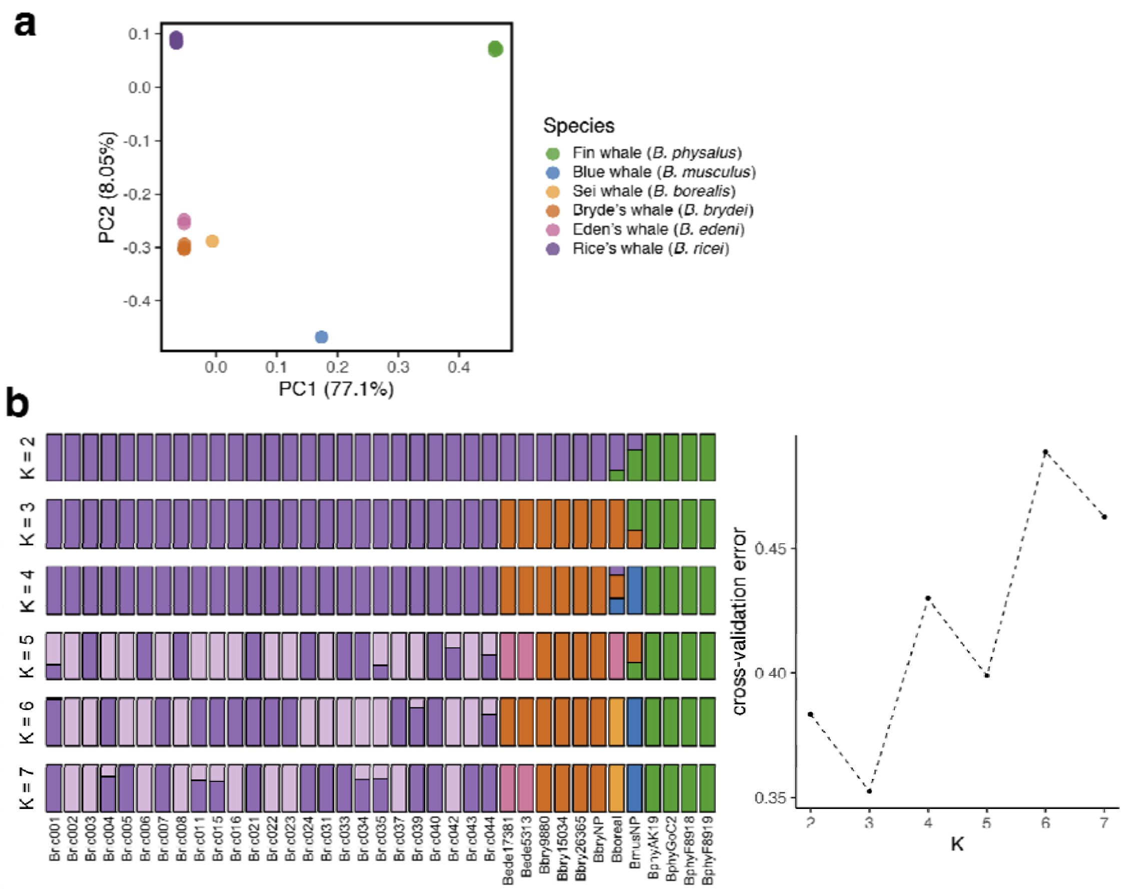

Supplemental Fig. 1 | Population structure. a, PCA with all individuals, minor allele frequency filter 5% **b,** ADMIXTURE analysis showing k values 2-7 and cross validation analysis

bioRxiv preprint doi: https://doi.org/10.64898/2026.06.15.732430; this version posted June 19, 2026. The copyright holder for this preprint (which was not certified by peer review) is the author/funder, who has granted bioRxiv a license to display the preprint in perpetuity. It is made available under a CC-BY 4.0 International license.

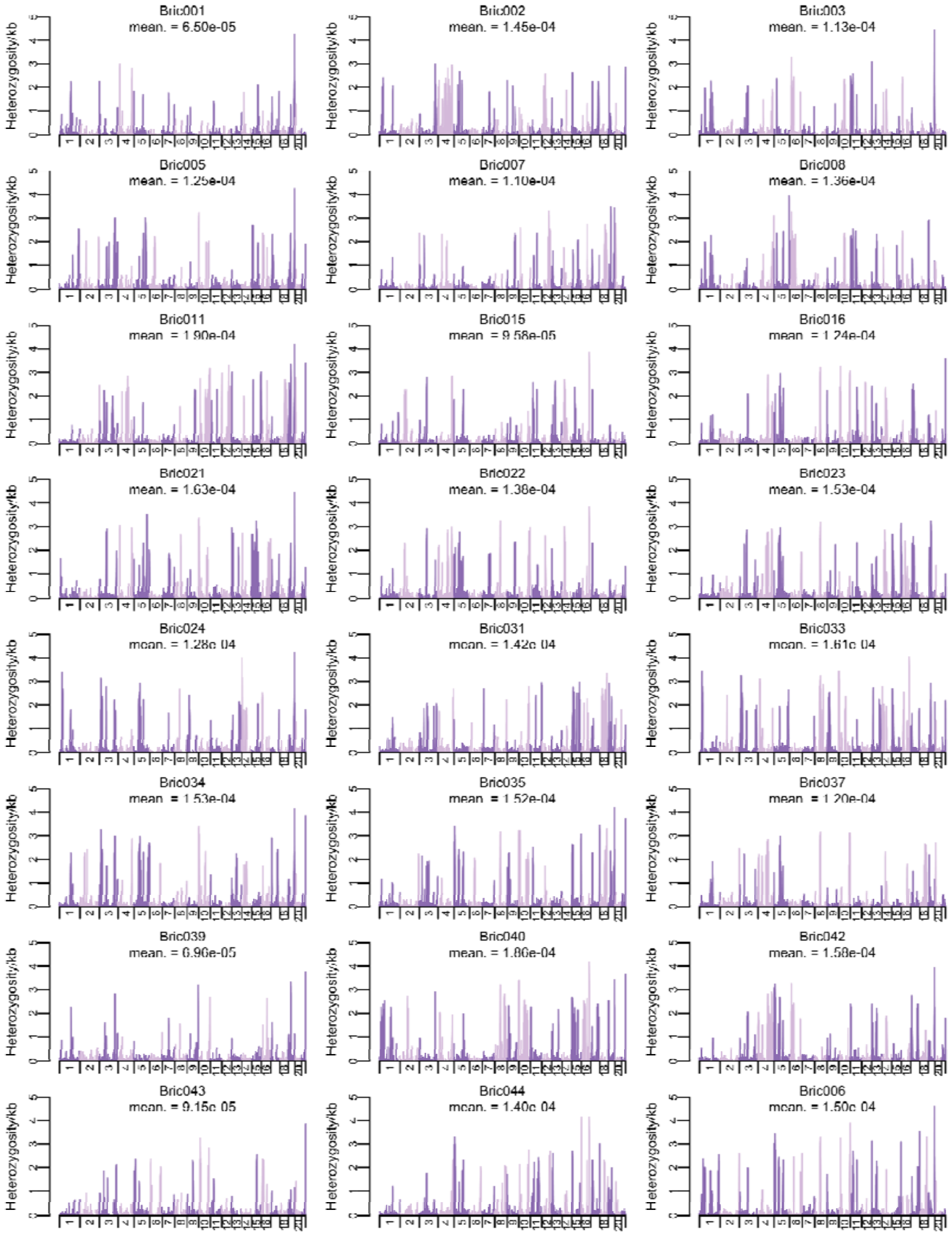

Supplemental Fig. 2 | Genome-wide heterozygosity patterns in Rice's whales.
Heterozygosity per kb in 1Mb windows in all sequenced Rice's whale individuals. Individual Bric004 is in the main Fig. 2

bioRxiv preprint doi: https://doi.org/10.64898/2026.06.15.732430; this version posted June 19, 2026. The copyright holder for this preprint (which was not certified by peer review) is the author/funder, who has granted bioRxiv a license to display the preprint in perpetuity. It is made available under a CC-BY 4.0 International license.

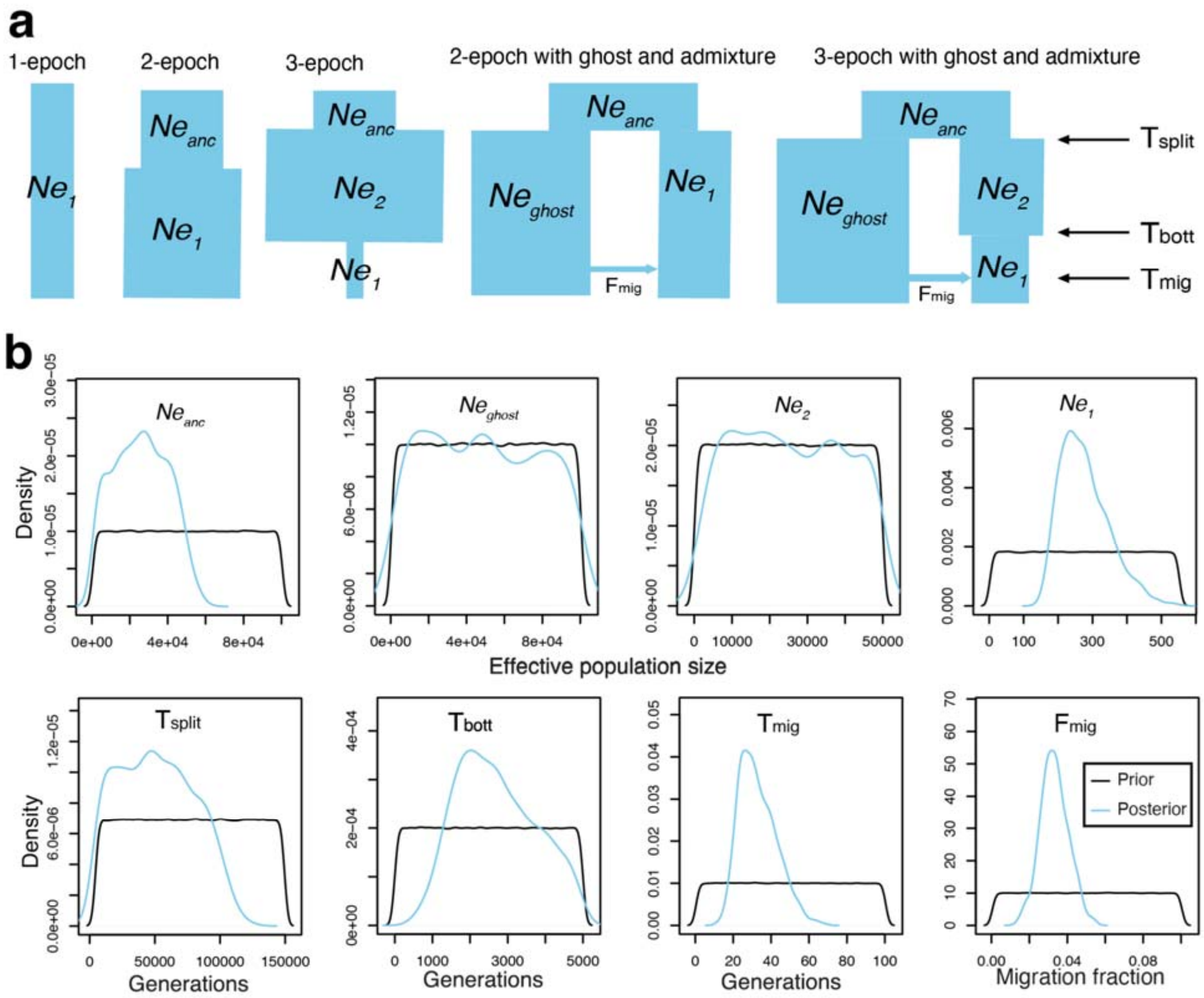

Supplemental Fig 3 | Demographic models and results for ABC inference. a, Pictorial representations of models tested in demographic inference (not to scale). **B,** Plots showing prior and posterior distributions of parameters for the "3-epoch ghost-admixture with a recent population decline" model from ABC. Posterior distributions are derived from the 1,000 best-fitting models out of 1 million total. Note that the N_{e1}, T_{mig}, and F_{mig} parameters are well resolved. The N_{eanc}, T_{split}, and T_{bott} parameters are moderately resolved, and the N_{eghost} and N_{e2} parameters are not resolved.

bioRxiv preprint doi: https://doi.org/10.64898/2026.06.15.732430; this version posted June 19, 2026. The copyright holder for this preprint (which was not certified by peer review) is the author/funder, who has granted bioRxiv a license to display the preprint in perpetuity. It is made available under a CC-BY 4.0 International license.

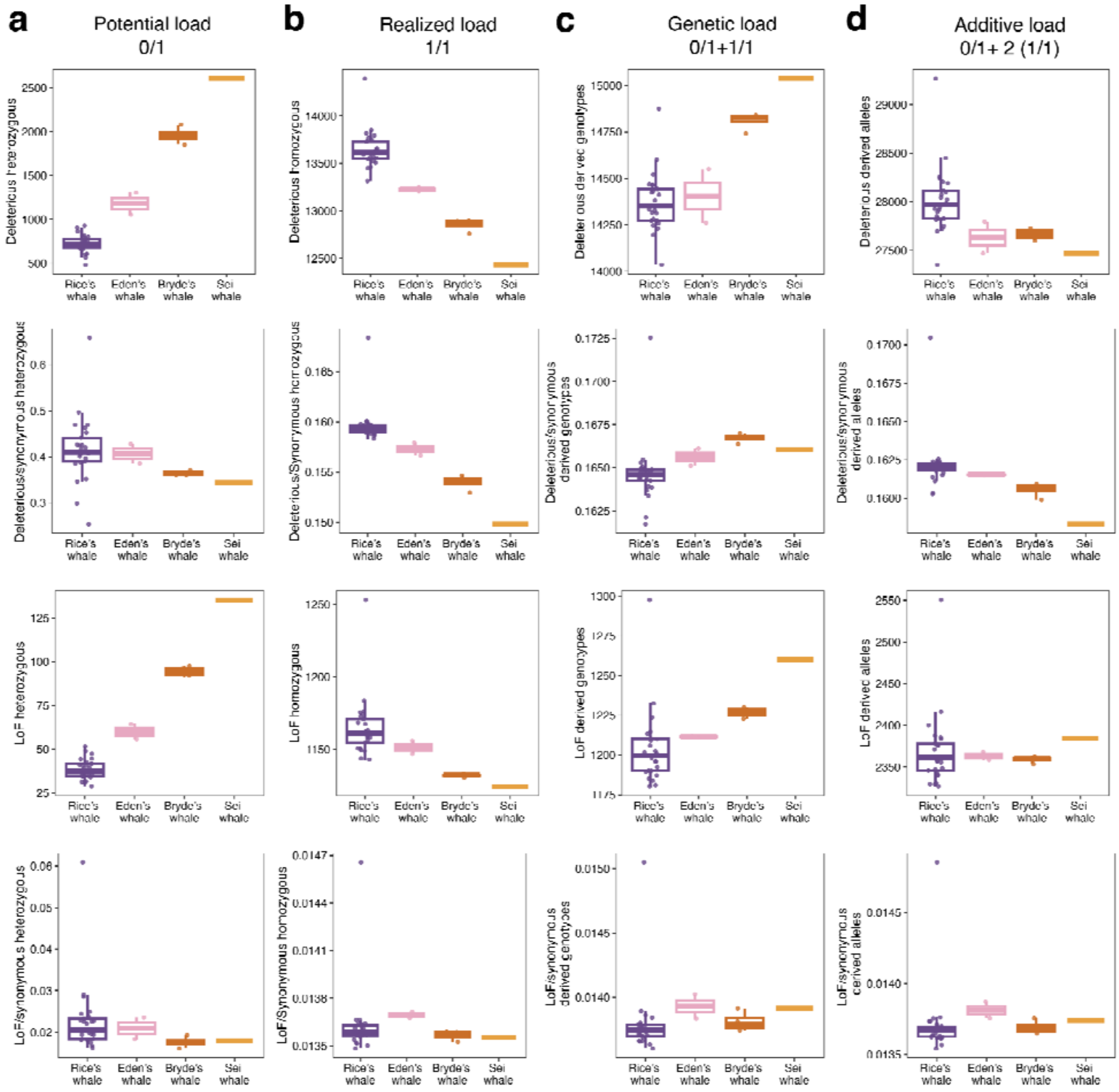

Supplemental Fig 4 | Deleterious and loss-of-function variant loads in baleen whales (extended). Deleterious (rows 1–2) and loss-of-function (LoF; rows 3–4) variant counts and ratios relative to synonymous variants are shown across four load categories in Rice's, Eden's, Bryde's, and sei whales. **a,** Potential load, calculated as the total number of heterozygous (0/1) derived variants. **b,** Realized load, calculated as the number of homozygous derived (1/1) variants. **c,** Genetic load, calculated as the combined count of heterozygous and homozygous derived variants (0/1) + (1/1). **d,** Additive load, calculated as the sum of all alternative alleles across individuals. Rows 1 and 3 show raw variant counts for deleterious and LoF variants, respectively. Rows 2 and 4 show the corresponding ratios normalized to synonymous variants.

bioRxiv preprint doi: https://doi.org/10.64898/2026.06.15.732430; this version posted June 19, 2026. The copyright holder for this preprint (which was not certified by peer review) is the author/funder, who has granted bioRxiv a license to display the preprint in perpetuity. It is made available under a CC-BY 4.0 International license.

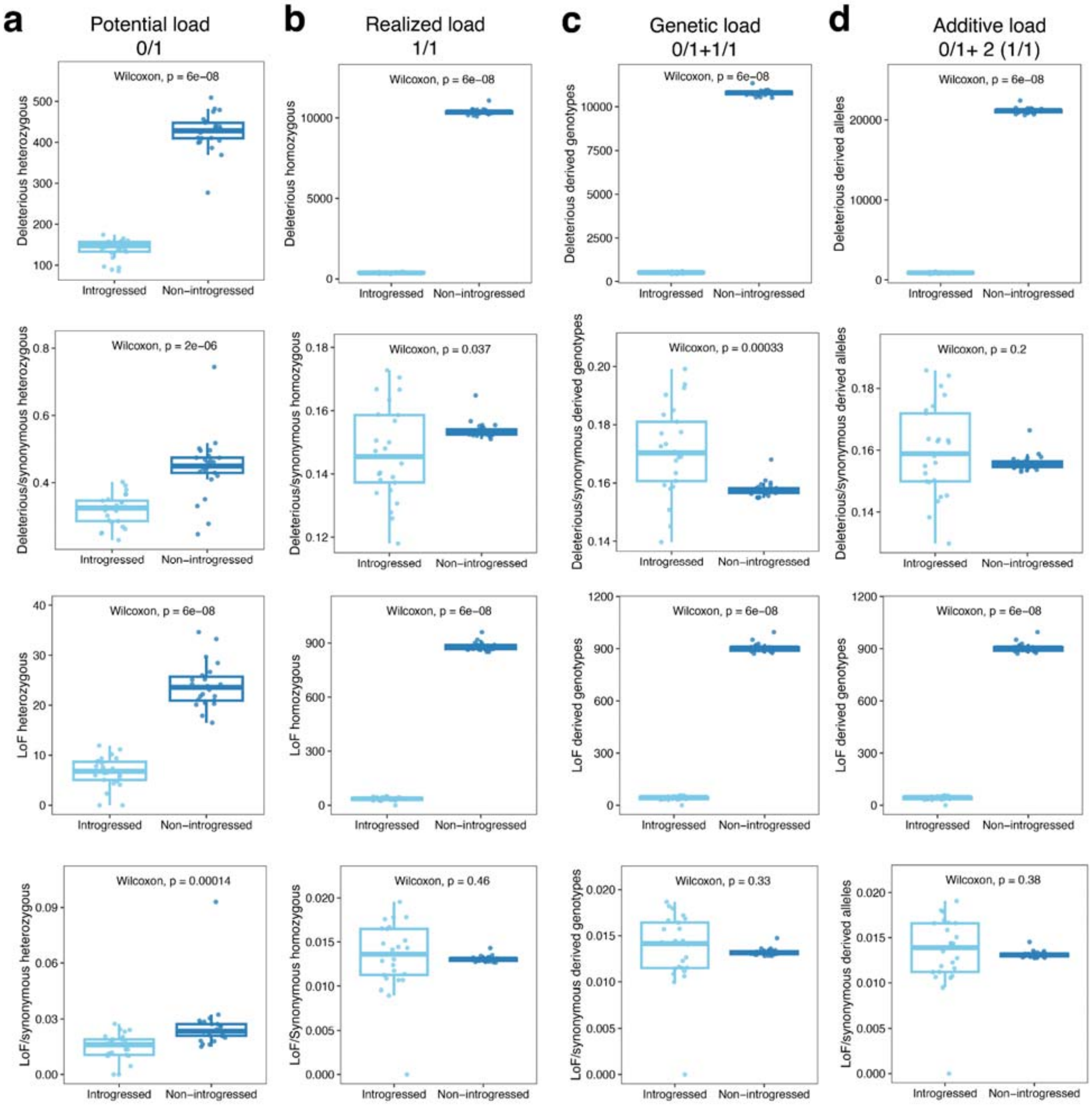

Supplemental Fig. 5 | Deleterious and loss-of-function variant loads in introgressed and non-introgressed regions of the Rice's whale genome (extended). Deleterious (rows 1–2) and loss-of-function (LoF; rows 3–4) variant counts and ratios relative to synonymous variants are shown across four load categories, partitioned by genomic context (introgressed vs. non-introgressed regions). **a,** Potential load, calculated as the total number of heterozygous (0/1) derived variants. **b,** Realized load, calculated as the number of homozygous derived (1/1) variants. **c,** Genetic load, calculated as the combined count of heterozygous and homozygous derived variants (0/1) + (1/1). **d,** Additive load, calculated as the sum of all alternative alleles across individuals. Rows 1 and 3 show raw variant counts for deleterious and LoF variants, respectively. Rows 2 and 4 show the corresponding ratios normalized to synonymous variants.

bioRxiv preprint doi: https://doi.org/10.64898/2026.06.15.732430; this version posted June 19, 2026. The copyright holder for this preprint (which was not certified by peer review) is the author/funder, who has granted bioRxiv a license to display the preprint in perpetuity. It is made available under a CC-BY 4.0 International license.

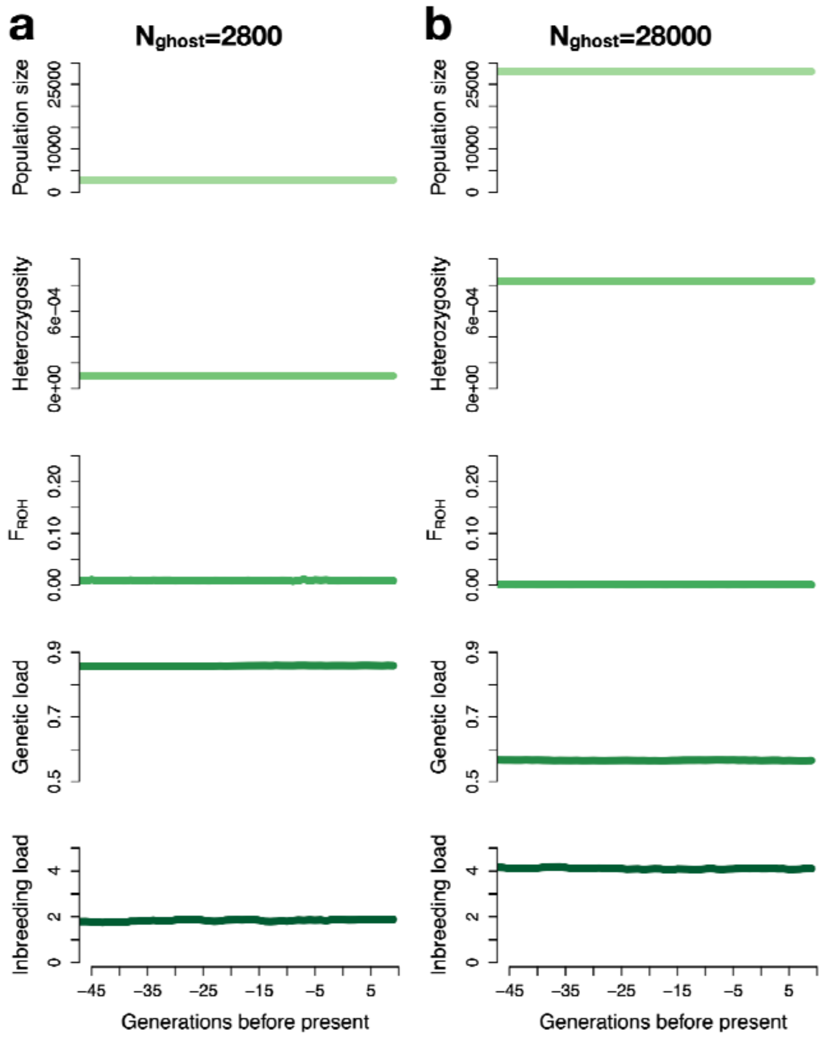

Supplemental Fig 6| Simulation results for the ghost population when assuming N_{ghost}=2800 or N_{ghost}=28000. For each simulated scenario, results are shown for 45 generations before present and projected 10 generations into the future. Top panel shows effective population size over time for the ghost population, second panel shows average observed heterozygosity, third panel shows average F_{ROH} for ROH>1Mb, fourth panel shows realized genetic load, and bottom panel shows inbreeding load calculated as the diploid number of lethal equivalents.
